# Clinical Benefit of Robotic-Assisted Total Knee Arthroplasty over Conventional Total Knee Arthroplasty When Using Mobile-Bearing Implants

**DOI:** 10.3390/medicina60071103

**Published:** 2024-07-06

**Authors:** Sang-Ho Han, Min-Soo Lee, Se-Hee Kong

**Affiliations:** 1 Department of Orthopedic Surgery, Daechan Hospital, 590 Inju-daero, Namdong-gu, Incheon 21570, Republic of Korea; doctortrust@naver.com (S.-H.H.); bluelms71@naver.com (M.-S.L.); 2 Daechan Sports Medical Research Center, 590 Inju-daero, Namdong-gu, Incheon 21570, Republic of Korea

**Keywords:** total knee replacement, joint range of motion, comparative study

## Abstract

*Background and Objectives:* As a treatment modality for advanced knee osteoarthritis, total knee arthroplasty is well established and has been performed on many patients over time. To improve surgical outcomes, fixed-bearing implant insertion with robotic-assisted TKA has been introduced; however, the insertion of mobile-bearing (MB) implants with the same method is challenging. The aim of this study was to compare the short-term postoperative follow-up outcomes of MB implant insertion using a robotic-assisted TKA system and conventional TKA. *Materials and Methods:* We investigated functional improvement in the knees of 60 patients who underwent the insertion of MB implants using either robotic-assisted TKA or conventional TKA. Isokinetic muscular function, range of motion, the Western Ontario and McMaster Universities Osteoarthritis Index score, visual analog scale (VAS) score, and Knee Society Score (KSS) were measured 6 months after surgery. The statistical analysis of outcome measurements was performed using the Mann–Whitney U test and the Wilcoxon signed-rank test. *Results:* Some isokinetic muscular functions, as well as Knee Society Scores (pain and function) and VAS scores, were significantly higher in patients who underwent MB insertion with robotic-assisted TKA than in those who underwent conventional TKA. *Conclusions:* When an MB implant is inserted using a robotic-assisted TKA system, a better surgical outcome can be expected.

## 1. Introduction

Total knee arthroplasty (TKA) is a well-established procedure with successful treatment outcomes, and for many years, it has been performed on many patients with end-stage knee osteoarthritis [1,2,3]. With the increase in the demand for TKA, there is increased interest in patient satisfaction with the surgery and the life span of artificial joints [4]. However, recent studies have shown that up to 20% of patients are dissatisfied with the maximum flexion of the knee joint following conventional TKA [1,5,6,7,8,9,10], which is due to the insecurity and inaccurate positioning of the artificial joint [4,11,12,13]. Therefore, a robotic-assisted TKA system, which improves security, positioning, and patient satisfaction, was created. The robotic-assisted TKA system uses 3D computed tomography to photograph and analyze the surgical site preoperatively, allowing the medical staff to select the range and angle of bone cutting and thus achieve accurate implant insertion. As a result, several clinical outcomes are improved, the complication rate is lowered, and the patient’s leg alignment is improved after surgery [14]. As several studies continue to provide evidence of the advantages of robotic TKA, the number of robotic-assisted TKA systems continues to increase.

To demonstrate the improved surgical outcomes of total knee arthroplasty (TKA), continuous advancements are being made in both surgical techniques and implant designs. Initially, fixed-bearing (FB) implants were predominantly used in TKA and robotic TKA procedures. TKA with FB implants has consistently shown favorable clinical outcomes. However, FB implants have been associated with drawbacks such as implant wear and loosening during surgery.

Mobile-bearing (MB) implants were introduced to address the limitations of fixed-bearing (FB) implants by enhancing conformity and minimizing contact stress [15].

Comparative studies have been conducted to validate the effectiveness of different implant designs. In the early studies, a majority of TKA procedures compared FB and MB implants. As time progressed, research on the application of FB implants in robot-assisted TKA systems also emerged. FB implants are employed in robot-assisted TKA systems due to their ability to enhance surgical accuracy by ensuring precise implant cutting. On the other hand, MB implants require proper balancing with the surrounding ligament tissues to maintain functional stability. Failure to achieve this balance may result in increased instability. The use of MB insertion is technically more challenging and requires a significant learning curve [16,17,18]. Consequently, integrating MB implants into robot-assisted TKA systems has been problematic. However, the research team of Curexo (Curexo, Inc., Seoul, Republic of Korea) and our hospital have recently developed a protocol to incorporate MB implants into robot-assisted TKA procedures. It is essential to evaluate the results of this introduction to enhance diversity in TKA surgical techniques and implant options.

A recent study similar to the current one has been conducted, but it utilized different navigation-assisted systems. Moreover, the objectives of these studies have been varied.

The purpose of this study is to compare the clinical outcomes of FB and MB implants applied in robotic-assisted TKA systems. Furthermore, this study hypothesizes that better results can be achieved by utilizing MB implants in robotic-assisted TKA.

## 2. Materials and Methods

### 2.1. Study Design

This was a retrospective cohort comparative study that compared the preoperative and 6-month postoperative outcomes of robotic-assisted and conventional TKA using an MB implant.

We compared patients who underwent TKA via a robotic-assisted TKA system and those who underwent conventional TKA between December 2020 and May 2022 at the Daechan Hospital in Incheon, South Korea. The required number of patients was calculated using G-Power 3.1. When calculated based on the *t*-test of G-Power, the total sample size was 52 people, with an error of 0.05, power of 0.8, and effect size of 0.8. Considering a 10% dropout rate, 60 patients (120 knees) were recruited, and they all ultimately participated in the study. These patients were mentally capable of completing the study; they understood the purpose of the research and voluntarily expressed their intention to participate in it. Patients underwent the procedure on both knees. The exclusion criteria were patients with repeated TKA (within 4 years), inflammatory arthritis (except for rheumatoid arthritis) after repeat surgery, or difficulty understanding and responding to the survey. The clinical data of the study participants are presented in Table 1.

This study was conducted upon obtaining approval from the institutional review board of our institution (No. P01-202104-21-006) and followed the STROBE reporting guidelines [19].

### 2.2. Surgery and Implant

**Figure 1:**
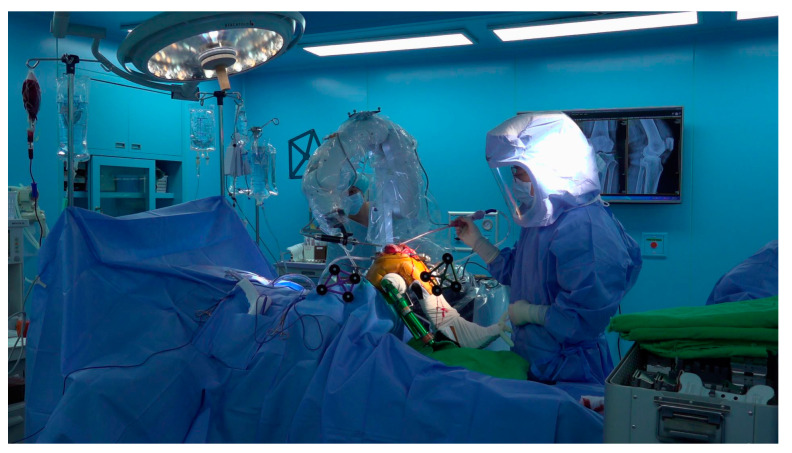
Robot-assisted TKA.

Two experienced orthopedic surgeons performed the surgeries in this study. They utilized an automated robotic-assisted TKA system (CUVIS-Joint Surgical^®^; Curexo, Inc., Seoul, Republic of Korea) with automatic cutting that serves as an assistant to primary surgeons during preoperative planning and surgery (Figure 1). The system uses a six-axis vertical multi-joint similar to those in a human arm to access a wide range of surgical areas and allow for natural movement. The implant used in this study was the Buechel–Pappas knee prosthesis (B-P Knee; Endotec, Orlando, FL, USA), a modified version of a low-contact-stress prosthesis.

The robot-assisted TKA system employs protocols from conventional TKA. Initially, computed tomography (CT scan) is performed, and the resulting 3D images are used to diagnose the patient’s condition and formulate a surgical plan. Virtual surgery is conducted to validate the plan and simulate surgical outcomes. During the procedure, the patient and robotic system are securely connected and fixed to prevent movement. Once the patient and the robot are fixed, the surgeon verifies the alignment between the patient’s 3D image and the actual surgical site. Final data checks ensure precise bone resection, matching the implant’s predetermined size, position, angle, and orientation from the surgical planning stage. The procedure concludes with the insertion and fixation of the MB implant.

### 2.3. Outcome Assessment

Outcomes before and 6 months after the surgery were evaluated based on isokinetic muscular function, range of motion (ROM), Knee Society Score (KSS) (pain and function), Western Ontario and McMaster Universities Osteoarthritis Index (WOMAC) score, and visual analog scale (VAS) score. The isokinetic muscular function test evaluated the muscular function using an isokinetic dynamometer (Biodex system 4 model; Biodex Medical Systems, Inc., Shirley, NY, USA). The score ranged from 0 to 100, and a higher score indicated poorer function. The peak torque of the quadriceps femoris and hamstring was measured at 60°/s and 180°/s of knee angular velocity, using VAS. Regarding passive ROM, professional exercise therapists and medical staff measured a maximum of 90° of movement at the hip joint by the knee flexor, using a standard portable protractor. The KSS evaluates pain in the knee and the function of the knee joints, scoring between 0 and 85 points, with a higher score indicating a better outcome. WOMAC was used to score pain in the knee, and stiffness evaluated the overall ease of moving the knee. Scoring assigned 0 points for no pain and 10 points for unbearable pain.

### 2.4. Statistical Analysis

Data analysis was performed using the SPSS statistical package version 22.0 (IBM Corp., Armonk, NY, USA). For each variable, the mean and standard deviation were calculated. To analyze the difference between preoperative and postoperative outcomes of MB applications in the robotic-assisted and conventional TKA groups, the Mann–Whitney U test, a nonparametric test, was performed. In addition, the Wilcoxon signed-rank test was used to examine the pre- and postoperative changes within groups. Due to the non-normal distribution of some missing data, a nonparametric test was performed. The statistical significance level was set at *p* < 0.05.

## 3. Results

The demographic characteristics of the patients were compared using the Mann–Whitney U test, the results of which are shown in Table 1. The mean ages of the robotic-assisted and conventional TKA groups were 70.57 ± 5.89 and 71.70 ± 6.47 years, respectively. There was no significant difference in weight and body mass index between the two groups. While the two groups showed a difference in height, this did not appear to have much impact on the results of the study.

To verify the difference in the dependent variables of the two surgical groups 6 months after surgery, the Mann–Whitney U test was performed. The preoperatively measured dependent variables showed no significant differences between the two groups, confirming pre-homogeneity between the groups. The results are presented in Table 2. There was a difference between the two groups regarding some of the dependent variables measured 6 months after surgery. In the case of ROM, there was a significant difference between the robotic and conventional TKA groups in both legs (right: 126.80 ± 6.43 vs. 121.60 ± 9.7, *p* < 0.05; and left: 127.60 ± 5.23 vs. 122.20 ± 139.58, *p* < 0.05), showing that the robot-assisted TKA system resulted in a greater improvement in ROM than conventional TKA.

There were significant differences in the pain and function scores of the KSS. The robot-assisted TKA group had improved outcomes with respect to both pain score (46.00 ± 8.17 vs. 41.00 ± 12.42, *p* < 0.05) and function score (75.00 ± 17.14 vs. 64.00 ± 15.53, *p* < 0.05). The VAS score was also lower (0.72 ± 0.79 vs. 1.85 ± 1.14, *p* < 0.05) in the robotic-assisted TKA group.

Although not all variables showed differences between the robotic-assisted TKA and conventional TKA groups, the application of MB implants seemed to have a more significant effect on the robotic-assisted TKA group than on the conventional TKA group, based on the ROM, pain and function scores of the KSS, and VAS score. However, since these points are premised on the effect of preoperative and postoperative improvements between the two groups, the Wilcoxon signed-rank test was performed to determine the difference in each of the two groups before and after surgery, and the results are presented in Table 3 and Table 4. The Rt Flex 60 values showed that the robotic-assisted TKA group had significant changes with regard to isokinetic muscle function before/after surgery (16.49 ± 8.78 vs. 16.18 ± 8.10, *p* < 0.05). The Rt Flex 180 values of the robotic-assisted TKA group before and after surgery were 13.03 ± 9.12 and 20.06 ± 9.23, respectively (*p* < 0.05), while those of the conventional TKA group were 12.34 ± 7.98 and 16.18 ± 8.10, respectively (*p* < 0.05); both groups had significantly different values before and after surgery. The degrees of change were 7.03 and 3.84 for the robotic-assisted and conventional TKA groups, respectively, and that of the robotic-assisted TKA group was higher. With regard to Lt Flex 180, there was a significant change only in the robotic-assisted TKA group (11.67 ± 7.65 vs. 18.30 ± 8.88, *p* < 0.05). In the case of isokinetic muscle function, significant changes were generally small, but function was improved with the robot-assisted TKA system.

**Figure 2:**
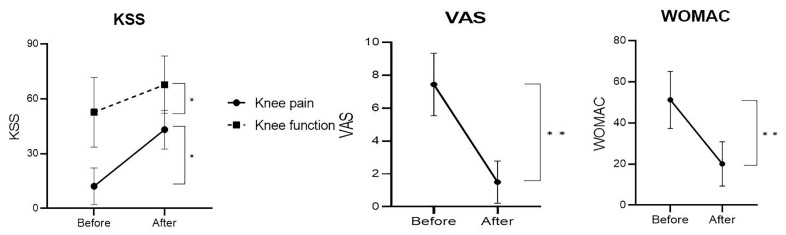
Clinical scores comparing the initial and final Knee Society Score, Visual Analogue Scale, and Western Ontario and McMaster Universities Osteoarthritis Index for knee pain and knee function., *p* < 0.05 *, *p* < 0.01 **.

In summary, our analysis showed significant changes in some functions in each of the robotic-assisted TKA and conventional TKA groups after surgery, and the changes were generally greater in the robotic-assisted TKA group. When the difference between the two groups was examined 6 months after surgery, the robotic-assisted TKA group showed significant improvement in some of the functions measured, including ROM, KSS, and VAS scores. Among them, the change in clinical scores is shown in Figure 2.

## 4. Discussion

The number of patients with osteoarthritis continues to increase as humans undergo inevitable physical deterioration, and this trend is expected to continue in the future. Consequently, the continuous development of total knee arthroplasty (TKA) aims to enhance the satisfaction level of osteoarthritis patients. Examples of this development include advancements in surgical methods (such as the introduction of robotic-assisted TKA systems), implant innovation (such as the application of mobile-bearing implants), and the use of cement for bone and implant fixation. Currently, extensive research is being conducted on various factors related to TKA surgeries.

However, the most significant advancement in TKA is the introduction of robot-assisted TKA systems and the use of new types of implants. The implementation of robot-assisted TKA has led to improved clinical outcomes, and the emergence of mobile-bearing (MB) implants has further enhanced knee mobility. MB implants are recognized for their potential benefits, particularly for patients under the age of 70 who desire an active lifestyle. Therefore, in this study, MB implants were inserted using a robot-assisted TKA system, and the surgical outcomes were compared to those of conventional TKA.

While comparative studies between fixed-bearing (FB) and mobile-bearing (MB) implants in conventional TKA have been frequently conducted, there is a lack of research specifically focusing on MB implant insertion using a robot-assisted TKA system. Most of the existing literature primarily explores the application of MB implants in conventional TKA and compares robot-assisted TKA to conventional TKA. In their study, Mahoney et al. reported that postoperative short-term follow-up outcomes of MB and FB implants using conventional TKA showed no statistically significant differences in WOMAC, the Short Form (12) (SF-12) score, or KSS until 2 years after surgery [20]. However, the MB implant group had slightly greater mean knee flexion at 6 months and 1 year after surgery [20]. Additionally, Artz et al. [21] established MB and FB implants using conventional TKA and examined the stability of the knee; they found that the FB group was significantly better according to self-reported outcomes at 1 and 2 years after surgery. Nevertheless, the two groups had similar results in terms of WOMAC and ROM [21]. In particular, studies that followed up patients more than 1 year after surgery showed better outcomes with FB implants; however, there was no difference between MB and FB implants with regard to WOMAC. In addition, there were cases where MB implants demonstrated better outcomes than FB implants, which were relatively short-term.

In a previous study, Jeon et al. [22] investigated the long-term clinical outcomes of patients who underwent robotic-assisted TKA or conventional TKA, in which an FB implant was used in both groups. The study found no clinical or radiological differences between the two groups; although the robotic-assisted TKA group tended to have accurate alignment in the lower extremities, there was no clear statistical difference. Additionally, Liow et al. [23] reported no difference in KSS function scores between the robotic-assisted and conventional TKA groups in their study. Moreover, it has been suggested that the robotic-assisted TKA system accurately restores the joint line, which could reduce the incidence of knee instability and improve clinical outcomes. In their study, Yang et al. [24] found no difference in WOMAC, ROM, and VAS scores between the use of the robotic-assisted TKA system and the conventional TKA method. In the literature that compared robotic-assisted TKA systems and conventional TKA, FB implants were used. The robotic-assisted TKA system tended to align the knees accurately, although it was difficult to clearly distinguish the difference between the robotic-assisted TKA system and the conventional TKA method in terms of functionality.

In this study, the correct alignment of the knee bones can be verified through ROM measurements. Both groups utilizing MB implants demonstrated postoperative improvement in ROM relative to preoperative scores; however, these improvements were not statistically significant. Notably, of the two groups, the group undergoing robot-assisted TKA with MB implants exhibited better ROM results. This result highlights the advantages of MB. The design allows free movement of the metal replacement under the plastic, potentially expanding knee ROM and facilitating movement in various positions. The improved outcomes are likely attributable to the combination of precise bone resection, a key advantage of the robot-assisted TKA system, and the accurate placement of MB implants.

In the study conducted by Lee et al. [25], a comparative study was performed to compare navigation-assisted versus conventional mobile-bearing total knee arthroplasty.

This study utilized a retrospective analysis to analyze the survival rate of implants and clinical outcomes, which is most similar to this study. The results showed no significant difference between mobile-bearing (MB) and fixed-bearing (FB) implants in terms of implant survival rate. There was no significant difference in WOMAC scores. The present findings are consistent with previous research, except for the KSS results. While earlier studies showed no significant difference in KSS between the two groups, this study demonstrated superior KSS outcomes in the robot-assisted MB implant group. This improvement may be attributed to more rapid functional improvements from direct activities such as walking and stair climbing. In addition, it is important to note that this study’s short-term follow-up of six months may have yielded results that differ from long-term outcomes. Furthermore, as implant survival rates were not investigated in this study, direct comparisons with previous research in this aspect are not feasible.

It was challenging to make a comprehensive comparison due to the variation in measurement methods between this study and previous studies. As a result of analyzing papers similar to this study, several research findings were obtained. However, generalization was difficult due to variations in measurement tools and follow-up periods across the studies.

Notably, there were limited papers found within our literature search that specifically analyzed the outcomes of fixed-bearing (FB) implants when applied to robot-assisted surgery with mobile-bearing (MB) implants. However, robot-assisted surgery was reported to exhibit greater accuracy in terms of lower extremity alignment and spacing balance. This finding aligns with the earlier statement that robotic-assisted TKA systems can enhance surgical accuracy. Furthermore, since there is scarce prior research on the application of MB implants in robot-assisted systems, more precise comparisons can only be made through continued research in this area.

This study has several limitations that should be addressed in future research. The limited awareness of MB implants among patients constrained the sample size. Future studies should aim to recruit a larger, more diverse patient population. The current sample’s gender imbalance, with a predominance of female participants, may have introduced bias. In addition, the prevalence of elderly patients necessitates the inclusion of younger subjects in subsequent analyses to enhance generalizability.

The six-month follow-up period, while practical given logistical constraints such as patient–hospital distance, is insufficient for assessing long-term outcomes. Extended follow-up studies are essential to evaluate the durability of results.

Intraoperative outcomes, blood loss, and radiological analyses were not assessed in this study. While the primary focus was on clinical outcomes of MB implant insertion rather than robotic surgery benefits, incorporating radiological data in future research would provide valuable insights, particularly in confirming lower extremity alignment accuracy. This addition would offer a more comprehensive evaluation of the robotic-assisted system’s efficacy.

Despite these limitations, this study yielded several noteworthy results. Firstly, although there has been limited research on the application of mobile-bearing (MB) implants in robot-assisted systems, this study utilized various measurements, such as range of motion (ROM) and muscle strength, which were not commonly used in previous studies. Secondly, data validity is ensured as we conducted an analysis over a reliable follow-up period. Thirdly, the study provides valuable data for patients to consider when selecting implants.

The results of this study are anticipated to serve as a foundation for future prospective long-term studies or other investigations that explore the application of MB implants in total knee arthroplasty (TKA).

## 5. Conclusions

In the short-term application of mobile-bearing (MB) implants in both the robotic TKA system and conventional TKA, significant improvements were observed in several functional aspects after surgery compared to preoperative measurements. Notably, the application of MB in the robot-assisted TKA system yielded better outcomes in certain functions such as range of motion (ROM), Knee Society Score (KSS), and Visual Analog Scale (VAS) scores, when compared to conventional TKA.

These findings contribute to the expanding body of knowledge on robotic TKA surgical options and suggest potential improvements in clinical outcomes. Future research should incorporate more comprehensive analyses, including the assessment of lower extremity alignment, the quantification of intraoperative blood loss, and long-term follow-up studies.

The potential application of robotic assistance extends beyond TKA to other orthopedic and surgical fields, warranting further investigation.

## Figures and Tables

**Table 1:** Baseline demographics on the robotic-assisted and conventional total knee arthroplasty groups.

Group (No. of Cases)	Conventional (n = 32)	Robotic-Assisted (n = 29)	Z	*p*-Value
Sex (Male/female)	0:32	3:26		0.102
Age (year, mean ± SD)	71.69 ± 6.57	70.62 ± 5.79	−0.079	0.430
Height (cm, mean ± SD)	154.09 ± 5.29	157.24 ± 7.32	1.622	0.105
Weight (kg, mean ± SD)	63.31 ± 9.82	63.52 ± 9.17	0.304	0.761
BMI (mean ± SD)	26.60 ± 3.46	25.64 ± 2.79	−0.845	0.398

Mann–Whitney U test, *p <* 0.05; BMI, body mass index; SD, standard deviation.

**Table 2:** Pre-and postoperative differences in the conventional group.

Variable			Conventional (n = 30)		
			Mean ± SD	*Z*	*p*-Value
Muscle torque at 60°/s	Rt Ext	Before	36.61 ± 13.86	−0.200	0.841
		After	36.76 ± 7.87		
	Rt Flex	Before	17.78 ± 10.46	−1.914	0.056
		After	21.25 ± 9.55		
	Lt Ext	Before	36.88 ± 16.06	−0.371	0.710
		After	35.67 ± 9.01		
	Lt Flex	Before	16.66 ± 9.86	−1.343	0.179
		After	19.48 ± 8.84		
Muscle torque at 180°/s	Rt Ext	Before	25.04 ± 10.69	−0.414	0.679
		After	25.26 ± 6.04		
	Rt Flex	Before	11.71 ± 8.14	−2.700	0.007 **
		After	16.18 ± 8.28		
	Lt Ext	Before	24.36 ± 10.74	−1.057	0.290
		After	26.56 ± 4.76		
	Lt Flex	Before	11.64 ± 7.59	−1.914	0.056
		After	15.15 ± 7.96		
R ROM		Before	121.94 ± 13.77	−0.052	0.958
		After	121.67 ± 9.96		
L ROM		Before	121.87 ± 14.58	−0.048	0.962
		After	121.88 ± 9.65		
KSS	Knee pain	Before	12.19 ± 10.08	−4.118	<0.001 **
		After	43.13 ± 10.61		
	Knee function	Before	52.66 ± 19.01	−2.933	0.003 *
		After	67.71 ± 15.67		
VAS		Before	7.44 ± 1.90	−4.300	<0.001 **
		After	1.50 ± 1.29		
WOMAC		Before	51.22 ± 13.90	−4.245	<0.001 **
		After	20.08 ± 10.77		

Wilcoxon signed-rank test, *p* < 0.05 *, *p* < 0.01 **; KSS, Knee Society Score; ROM, range of motion; SD, standard deviation; VAS, visual analog scale; WOMAC, Western Ontario and McMaster Universities Osteoarthritis Index.

**Table 3:** Clinical comparison results in the robotic-assisted and conventional total knee arthroplasty groups after six months.

Variable		Group	Cases	Mean ± SD	*Z*	*p*-Value
Muscle torque at 60°/s	Rt Ext	Robotic	30	38.79 ± 13.27	−0.369	0.712.
		Conventional	30	36.76 ± 7.87		
	Rt Flex	Robotic	30	22.86 ± 13.27	0.049	0.961
		Conventional	30	21.25 ± 9.55		
	Lt Ext	Robotic	30	39.39 ± 22.80	−0.534	0.593
		Conventional	30	35.67 ± 9.01		
	Lt Flex	Robotic	30	23.84 ± 15.11	0.806	0.420
		Conventional	30	19.48 ± 8.84		
Muscle torque at 180°/s	Rt Ext	Robotic	30	27.04 ± 12.58	0.000	1.000
		Conventional	30	25.26 ± 6.04		
	Rt Flex	Robotic	30	19.31 ± 10.98	0.631	0.528
		Conventional	30	16.18 ± 8.28		
	Lt Ext	Robotic	30	29.13 ± 15.36	−0.456	0.648
		Conventional	30	26.56 ± 4.76		
	Lt Flex	Robotic	30	18.46 ± 11.56	0.573	0.567
		Conventional	30	15.15 ± 7.96		
ROM	R ROM	Robotic	30	128.46 ± 6.13	2.651	0.008 *
		Conventional	30	121.67 ± 9.96		
	L ROM	Robotic	30	127.69 ± 5.14	2.426	0.015 *
		Conventional	30	121.88 ± 9.65		
KSS	Knee pain	Robotic	30	46.73 ± 6.47	2.251	0.024 *
		Conventional	30	43.13 ± 10.61		
	Knee function	Robotic	30	76.35 ± 16.47	1.991	0.046 *
		Conventional	30	67.71 ± 15.67		
VAS		Robotic	30	0.73 ± 0.78	−2.287	0.022 **
		Conventional	30	1.50 ± 1.29		
WOMAC		Robotic	30	17.65 ± 7.62	−0.574	0.566
		Conventional	30	20.08 ± 10.77		

Mann–Whitney U test, *p* < 0.05 *; *p* < 0.01 **; KSS, Knee Society Score; ROM, range of motion; SD, standard deviation; VAS, visual analog scale; WOMAC, Western Ontario and McMaster Universities Osteoarthritis Index.

**Table 4:** Pre- and postoperative differences in the robotic-assisted group.

Variable			Robotic-Assisted (n = 30)		
			Mean ± SD	*Z*	*p*-Value
Muscle torque at 60°/s	Rt Ext	Before	36.47 ± 11.93	−0.411	0.681
		After	38.79 ± 13.27		
	Rt Flex	Before	16.34 ± 8.56	−2.016	0.044 *
		After	22.86 ± 13.27		
	Lt Ext	Before	39.99 ± 16.37	−0.093	0.926
		After	39.39 ± 22.80		
	Lt Flex	Before	16.14 ± 8.09	−1.979	0.048 *
		After	23.84 ± 15.11		
Muscle torque at 180°/s	Rt Ext	Before	26.90 ± 10.40	−0.161	0.872
		After	27.04 ± 12.58		
	Rt Flex	Before	13.51 ± 8.38	−2.166	0.030 *
		After	19.31 ± 10.98		
	Lt Ext	Before	27.71 ± 11.02	−0.093	0.926
		After	29.13 ± 15.36		
	Lt Flex	Before	11.93 ± 7.46	−2.315	0.021 *
		After	18.46 ± 11.56		
R ROM		Before	124.31 ± 22.35	−0.071	0.944
		After	128.46 ± 6.13		
L ROM		Before	124.83 ± 22.66	−1.029	0.303
		After	127.69 ± 5.14		
KSS	Knee pain	Before	16.21 ± 12.23	−4.391	<0.001 **
		After	46.73 ± 6.47		
	Knee function	Before	52.93 ± 20.11	−3.752	<0.001 **
		After	76.35 ± 16.47		
VAS		Before	6.79 ± 1.88	−4.478	<0.001 **
		After	0.73 ± 0.78		
WOMAC		Before	49.83 ± 11.99	−4.459	<0.001 **
		After	17.65 ± 7.62		

Wilcoxon signed-rank test, *p* < 0.05 *, *p* < 0.01 **; KSS, Knee Society Score; ROM, range of motion; SD, standard deviation; VAS, visual analog scale; WOMAC, Western Ontario and McMaster Universities Osteoarthritis Index.

## Data Availability

The datasets analyzed during the current study are publicly available (https://doi.org/10.6084/m9.figshare.25906456 (accessed on 13 April 2021)).
